# Congenital Disorders of Deficiency in Glycosaminoglycan Biosynthesis

**DOI:** 10.3389/fgene.2021.717535

**Published:** 2021-09-03

**Authors:** Shuji Mizumoto, Shuhei Yamada

**Affiliations:** Department of Pathobiochemistry, Faculty of Pharmacy, Meijo University, Nagoya, Japan

**Keywords:** chondroitin sulfate, dermatan sulfate, heparan sulfate, proteoglycan, glycosaminoglycan, connective tissue disorder, skeletal disorder, skin disorder

## Abstract

Glycosaminoglycans (GAGs) including chondroitin sulfate, dermatan sulfate, and heparan sulfate are covalently attached to specific core proteins to form proteoglycans, which are distributed at the cell surface as well as in the extracellular matrix. Proteoglycans and GAGs have been demonstrated to exhibit a variety of physiological functions such as construction of the extracellular matrix, tissue development, and cell signaling through interactions with extracellular matrix components, morphogens, cytokines, and growth factors. Not only connective tissue disorders including skeletal dysplasia, chondrodysplasia, multiple exostoses, and Ehlers-Danlos syndrome, but also heart and kidney defects, immune deficiencies, and neurological abnormalities have been shown to be caused by defects in GAGs as well as core proteins of proteoglycans. These findings indicate that GAGs and proteoglycans are essential for human development in major organs. The glycobiological aspects of congenital disorders caused by defects in GAG-biosynthetic enzymes including specific glysocyltransferases, epimerases, and sulfotransferases, in addition to core proteins of proteoglycans will be comprehensively discussed based on the literature to date.

## Introduction

Glycosaminoglycans (GAGs), including chondroitin sulfate (CS), dermatan sulfate (DS), and heparan sulfate (HS), are linear polysaccharides that are covalently attached to core proteins, forming proteoglycans (PGs). PGs are ubiquitously distributed on the cell surface and in the extracellular matrix (Iozzo, 1998; Bishop et al., 2007; Thelin et al., 2013; Mizumoto et al., 2015a). GAGs are critically involved in a variety of biological functions including cell adhesion, cellular signaling, and the architecture of the extracellular matrix. A large number of studies have reported analyses of GAGs from various types of cells, nematodes, fruit flies, zebrafish, chicks, and mice (Häcker et al., 2005; Bülow and Hobert, 2006; Mizumoto et al., 2014). After the mapping of the of human genome, congenital disorders caused by defects in GAG biosynthesis have been revealed by many research groups (Mizumoto et al., 2013, 2014, 2015b; Mizumoto, 2018). This review provides a comprehensive overview of genetic disorders with symptoms affecting various areas of the body such as bone, skin, brain, heart, and immune system caused by defects in the biosynthesis of GAG side chains of PGs.

## Biosynthetic Pathway of Glycosaminoglycans

### Biosynthesis of Donor Substrates for GAGs and Transporters of Uridine 5′-Diphosphate-Sugars, Sulfate Ions, and 3′-Phosphoadenosine 5′-Phosphosulfate

**FIGURE 1:**
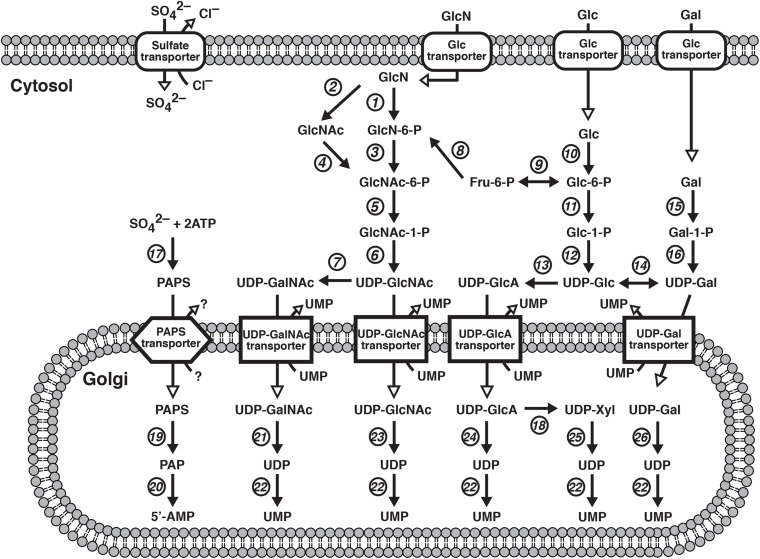
Biosynthetic pathways of UDP-sugars and PAPS. 1, Hexokinase; 2, GlcNH_2_ acetyltransferase; 3, GlcNH_2_ 6-phosphate *N*-acetyltransferase; 4, GlcNAc kinase; 5, GlcNAc phosphomutase; 6, UDP-GlcNAc pyrophosphorylase: 7, UDP-GalNAc 4-epimerase; 8, glutamine-fructose-6-phosphate aminotransferase; 9, Glc-6-phosphate isomerase; 10, hexokinase; 11, phosphoglucomutase; 12, UDP-Glc pyrophosphorylase; 13, UDP-Glc dehydrogenase; 14, UDP-Glc 4-epimerase; 15, galactokinase; 16, Gal-1-phosphate uridylyltransferase; 17, PAPS synthase; 18, UDP-GlcA decarboxylase; 19, sulfotransferases; 20, adenosine-3′, 5′-bisphosphate 3′-phosphatase; 21, GalNAc transferases; 22, nucleoside 5′-diphosphate phosphatase; 23, GlcNAc transferases; 24, GlcA transferases; 25, Xyl transferases; 26, Gal transferases.

Most glycosyltransferases and sulfotransferases utilize donor substrates such as uridine 5′-diphosphate (UDP)-sugars and 3′-phosphoadenosine 5′-phosphosulfate (PAPS), respectively. The nucleotide sugars, UDP-Glc, UDP-GlcA, UDP-GlcNAc, UDP-GalNAc, UDP-Gal, and UDP-Xyl, where Glc, GlcA, GlcNAc, GalNAc, Gal, and Xyl, represent D-glucose, D-glucuronic acid, *N*-acetyl-D-glucosamine, *N*-acetyl-D-galactosamine, D-galactose, and D-xylose, respectively, are produced predominantly from Glc, D-glucosamine (GlcN), and D-Gal (Figure 1). UDP-GlcA is formed by the action of the UDP-Glc dehydrogenase on UDP-Glc in the cytosol (Spicer et al., 1998). UDP-Xyl is formed by the action of UDP-GlcA decarboxylase/UDP-xylose synthase in the endoplasmic reticulum and Golgi apparatus (Moriarity et al., 2002). These UDP-sugars mainly synthesized in the cytosol, except for UDP-Xyl, are incorporated into the endoplasmic reticulum and Golgi lumen through nucleotide sugar transporters (Figure 1; Berninsone and Hirschberg, 2000).

Various GAG sulfotransferases catalyze the transfer of the sulfate group from the donor substrate, PAPS, to respective acceptor substrates (Kusche-Gullberg and Kjellén, 2003). PAPS is formed from adenosine 5′-triphosphate (ATP) and inorganic sulfate, which is incorporated into the cytosol through the sulfate transporter at the plasma membrane (Figure 1; Hästbacka et al., 1994). PAPS synthase (PAPSS) is a dual enzyme containing ATP sulfurylase and adenosine 5′-phosphosulfate kinase domains at C- and N-terminals, respectively (Venkatachalam, 2003).

### Glycosaminoglycan Backbones

**FIGURE 2:**
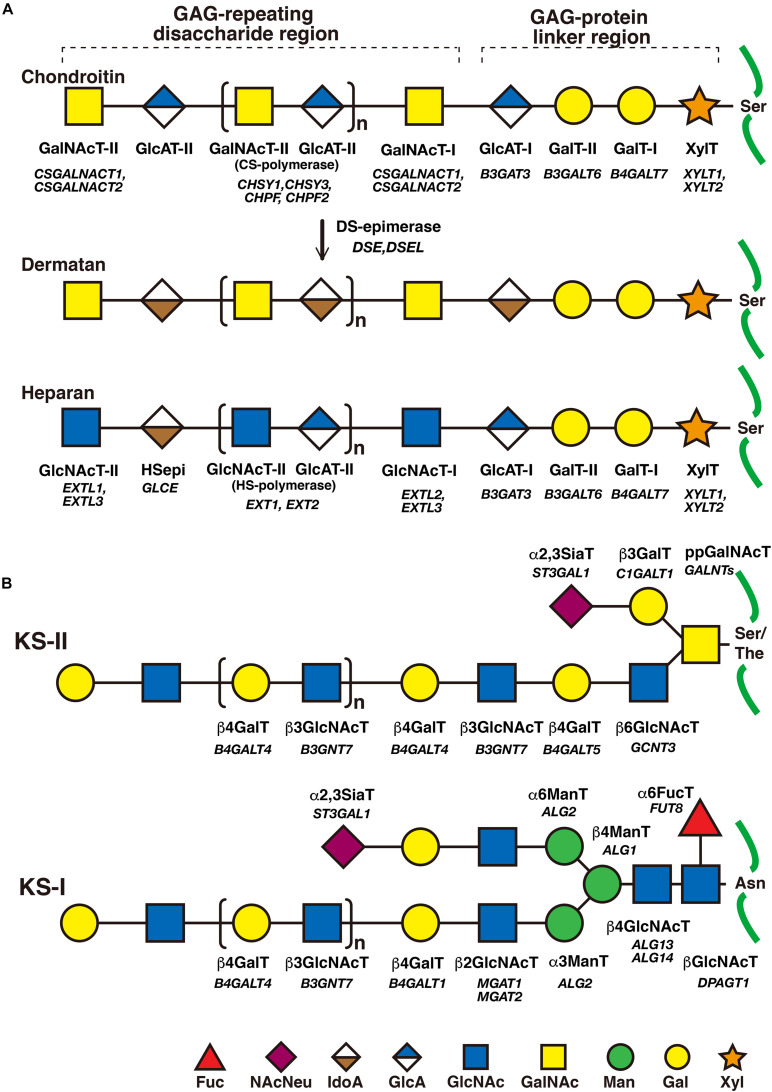
The biosynthetic assembly of GAGs. **(A)** Schematic presentation of the biosynthesis of CS, DS, and HS. Following the synthesis of specific core proteins of proteoglycans, the common GAG-linker region tetrasaccharide (CS, DS, and HS except for KS), GlcA-Gal-Gal-Xyl-*O*-, is built up by the consecutive addition of each sugar moiety from the respective UDP-sugar, including UDP-Xyl, UDP-Gal, or UDP-GlcA, by XylT, GalT-I, GalT-II, and GlcAT-I. The first GalNAc residue is then transferred to the GlcA residue at the non-reducing end in the linker region by GalNAcT-I, which initiates the construction of the chondroitin backbone. The repeating disaccharide region (chondroitin backbone of CS), [-3GalNAcβ1–4GlcAβ1-]_*n*_, is elongated by alternate additions of GlcA and GalNAc residues from UDP-GlcA and UDP-GalNAc catalyzed by CS-GlcAT-II and GalNAcT-II activities, respectively. After or during the formation of the chondroitin backbone, DSE converts GlcA into IdoA by epimerizing the C-5 carboxy group, resulting in the formation of the dermatan backbone of DS, [-3GalNAcβ1–4IdoUAα1-]_*n*_. Alternatively, the first GlcNAc residue is transferred to the GlcA residue at the non-reducing end in the linker region by GlcNAcT-I, which initiates the construction of the heparan backbone. The repeating disaccharide region (heparan backbone of HS), [-4GlcNAcα1–4GlcAβ1-]_*n*_, is elongated by alternate additions of the GlcA and GlcNAc residues from UDP-GlcA and UDP-GlcNAc catalyzed by HS-GlcAT-II and GlcNAcT-II activities, respectively. **(B)** Schematic presentation of the biosynthesis of KS. After the formation of the linkage region of KS, *O*- and *N*-linked oligosaccharides, the repeating disaccharide region, [-4GlcNAcβ1–3Galβ1-]_*n*_, is elongated by alternate additions of the Gal and GlcNAc residues from UDP-Gal and UDP-GlcNAc catalyzed by β4GalT and β3GlcNAcT activities, respectively. Each enzyme and its coding gene are described under the respective sugar symbols from the top of each line.

The biosynthesis of the linker region tetrasaccharide in CS, DS, and HS, but not keratan sulfate (KS) is initiated by the transfer of a xylose (Xyl) from UDP-Xyl to the specific serine residues on the core proteins of PGs by xylosyltransferase (XylT) encoded by *XYLT1* and *XYLT*2 in the endoplasmic reticulum (Table 1; Götting et al., 2000; Pönighaus et al., 2007). Thereafter, galactosyltransferase-I (GalT-I), galactosyltransferase-II (GalT-II), and glucuronosyltransferase-I (GlcAT-I), which are encoded by *B4GALT7*, *B3GALT6*, and *B3GAT3*, respectively, transfer two galactoses (Gals) and a glucuronic acid (GlcA) from UDP-Gal and UDP-GlcA to the Xyl residues in the Golgi apparatus (Figure 2A; Kitagawa et al., 1998; Almeida et al., 1999; Okajima et al., 1999; Bai et al., 2001). Modifications in the linker region tetrasaccharide, including the 2-*O*-phosphorylation of the Xyl residue as well as sulfation at the C-6 position of the first Gal and at C-4 or C-6 of the second Gal residue, have been reported (Sugahara and Kitagawa, 2000). These modifications are catalyzed by GAG-Xyl 2-*O*-kinase, Xyl 2-*O*-phosphatase, and Gal-6-*O*-sulfotransferase encoded by *FAM20B*, *ACPL2*, and *CHST3*, respectively (Kitagawa et al., 2008; Koike et al., 2009, 2014), and affect the glycosyltransferase reactions of GalT-I and GlcAT-I *in vitro*, which may regulate the biosynthesis of GAG chains (Gulberti et al., 2005; Tone et al., 2008).

**TABLE 1:** Congenital disorders of GAG-linker region tetrasaccharide deficiency*.

Gene	Protein**	Chromosomal location	MIM number	Name of disorder	Clinical hallmarks
*XYLT1*	XylT1	16p12.3	615777 608124	Desbuquios dysplasia type 2; Short stature syndrome	A short stature, joint laxity, advanced carpal ossification, a flat face with prominent eyes, and hand anomalies.
			300881	Baratela-Scott syndrome	A short stature, patellar dislocation, short tubular bones, mild metaphyseal changes, brachymetacarpalia with stub thumbs, short femoral necks, shallow acetabular roofs, platyspondyly, flattened midface with broad nasal bridge, cleft palate, bifid uvula, and synophrys.
*XYLT2*	XylT2	17q21.33	605822 608125	Spondyloocular syndrome	Osteoporosis, cataracts, sensorineural hearing loss, and mild learning defects.
*B4GALT7*	GalT-I	5q35.3	130070 604327	Ehlers-Danlos syndrome spondylodysplastic type 1; Ehlers-Danlos syndrome progeroid type 1; Ehlers-Danlos syndrome with a short stature and limb anomalies	Developmental delay, aged appearance, a short stature, craniofacial dysmorphism, defective wound healing with atrophic scars, generalized osteopenia, joint hypermobility, radioulnar synostosis, severe hypermetropia, blue sclerae, refractive, errors, corneal clouding, strabismus, nystagmus, cataracts, glaucoma, and retinal abnormalities.
				Larsen of Reunion Island syndrome	Multiple dislocations, hyperlaxity, dwarfism, and distinctive facial features.
*B3GALT6*	GalT-II	1p36.33	615349 615291	Ehlers-Danlos syndrome spondylodysplastic type 2; Ehlers-Danlos syndrome progeroid type 2	A short stature, joint laxity and dislocation, facial dysmorphism, joint contractures, severe bone fragility with multiple fractures, spondyloepimetaphyseal dysplasia, and intellectual disability.
			271640	Spondyloepimetaphyseal dysplasia with joint laxity type 1	Spatulate fingers with short nails, hip dislocation, elbow contracture, clubfeet, and mild craniofacial dysmorphism including prominent eyes, blue sclera, a long upper lip, and a small mandible with cleft palate.
*B3GAT3*	GlcAT-I	11q12.3	245600 606374	Multiple joint dislocations, a short stature, craniofacial dysmorphism with or without congenital heart defects; Larsen-like syndrome B3GAT3 type; B3GAT3-related disorder with dislocation and congenital heart defects	Joint dislocations mainly affecting the elbow, congenital heart defects (a bicuspid aortic valve and aortic root dilatation), osteoporosis, hypotonia, joint laxity, fractures, scoliosis, a biscuspid aortic valve, and myopia.
				B3GAT3-related disorder with cutis laxa and bone fragility	Spondyloepimetaphyseal dysplasia, cutis laxa, osteoporosis, fractures, multiple bony chondromas, and a short stature.
				B3GAT3-related disorder with craniosynostosis and bone fragility	Craniosynostosis, midface hypoplasia with proptosis, long tapered fingers, elbow joint contracture due to radioulnar synostosis, and an equinovarus position of the feet.
			264180	Pseudodiastrophic dysplasia	Prenatal manifestation, early lethality, short-limbed short stature at birth, facial dysmorphism, and distinctive skeletal abnormalities including short ribs, mild to moderate platyspondyly, broad ilia with flaring, increased acetabular angle, shortened long bones with metaphyseal flaring, elongation of the proximal and middle phalanges with subluxation of the proximal interphalangeal joints, subluxation of the elbow, and talipes equinovarus.
*FAM20B*	Xylosylkinase (GXK1)	1q25.2	611063	Severe (lethal) neonatal short limb dysplasia with multiple dislocations	Neonatal lethality, mid-face hypoplasia, thoracic hypoplasia with respiratory failure, a very short stature with mesomelic shortening of the limbs, and multiple dislocations of large joints.

*, the table was cited from a reference (Mizumoto, 2018) with modifications. **, or enzymatic activity. MIM, Mendelian inheritance in man; XYLT, xylosyltransferase; B4GALT7, xylosylprotein beta 1,4-galactosyltransferase 7; B3GALT6, beta 1,3-galactosyltransferase 6; B3GAT3, beta 1,3-glucuronyltransferase 3; FAM20B, Family with sequence similarity 20 member B.

The CS-repeating disaccharide region, [GlcA–*N*-acetyl-galactosamine (GalNAc)]_*n*_, is constructed by chondroitin synthases (CHSYs) including CHSY1, CHSY3, chondroitin polymerizing factor (CHPF), CHPF2, CS *N*-acetylgalactosaminyltransferase 1 (CSGALNACT1), and CSGALNACT2 using UDP-GlcA and UDP-GalNAc as donor substrates in the Golgi apparatus (Figure 2A and Table 2; Kitagawa et al., 2001, 2003; Uyama et al., 2002, 2003; Izumikawa et al., 2007, 2008).

**TABLE 2:** Congenital disorders of CS and DS deficiencies*.

Gene	Protein	Chromosomal location	MIM number	Name of disorder	Clinical hallmarks
*CHSY1*	ChSy1 (GalNAcT-II, CS-GlcAT-II)	15q26.3	605282 608183	Temtamy preaxial brachydactyly syndrome	A short stature, bilateral, symmetric preaxial brachydactyly and hyperphalangism of the digits, facial dysmorphism, dental anomalies, sensorineural hearing loss, delayed motor and mental development, and growth retardation.
*CSGALNACT1*	CSGalNAcT1 (GalNAcT-I, GalNAcT-II)	8p21.3	616615	Skeletal dysplasia, mild, with joint laxity and advanced bone age	Brachydactyly, joint laxity, mild facial dysmorphism consisting of midface hypoplasia, a flat nasal bridge, short nose, anteverted nares, a long philtrum, and microretrognathia.
*DSE*	DS-epimerase-1	6q22.1	615539 605942	Ehlers-Danlos syndrome musculocontractural type 2	Characteristic facial features, congenital contracture of the thumbs and feet, hypermobility of the finger, elbow, and knee joints, atrophic scarring of the skin, and myopathy.
*DSEL*	DS-epimerase-2	18q22.1	611125	Bipolar disorder; Depressive disorder	Alternating episodes of depression and mania or hypomania.
				Diaphragmatic hernia; Microphthalmia	Diaphragmatic hernia, respiratory difficulties, and microphthalmia.
*CHST3*	C6ST-1	10q22.1	143095 603799	Spondyloepiphyseal dysplasia with congenital joint dislocations; Spondyloepiphyseal dysplasia Omani type; Chondrodysplasia with multiple dislocations Megarbane type; Humerospinal dysostosis; Larsen syndrome autosomal recessive type; Desbuquois syndrome	A short stature, severe kyphoscoliosis, osteoarthritis (elbow, wrist, and knee), secondary dislocation of large joints, rhizomelia, fusion of carpal bones, mild brachydactyly, metacarpal shortening, ventricular septal defect, mitral and tricuspid defects, aortic regurgitation, and deafness.
*CHST11*	C4ST-1	12q23.3	610128 618167	Osteochondrodysplasia, brachydactyly, and overlapping malformed digits	Brachydactyly, overriding digits and clinosymphalangism in hands and feet, and syndactyly and hexadactyly in feet, scoliosis, dislocated patellae, and fibulae and pectus excavatum.
*CHST14*	D4ST-1	15q15.1	601776 608429	Ehlers-Danlos syndrome musculocontractural type 1; Ehlers-Danlos syndrome, type VIB; Adducted thumb-clubfoot syndrome	Craniofacial dysmorphism, multiple contractures, progressive joint and skin laxities, multisystem fragility-related manifestations, contracture of the thumbs and feet, defects of the heart, kidneys, and intestines.
*UST*	CS/DS2ST	6q25.1	610752	Multiple congenital anomalies of the heart and central nervous system	Growth failure, congenital heart defects, and underdeveloped cerebellar vermis, abnormal cutaneous elasticity, and joint laxity.

*, the table was cited from a reference (Mizumoto, 2018) with modifications. CHSY, chondroitin sulfate synthase; CHPF, chondroitin polymerizing factor; CSGALNACT, chondroitin sulfate N-acetylgalactosaminyltransferase; DSE, dermatan sulfate epimerase; DSEL, dermatan sulfate epimerase-like; CHST, carbohydrate sulfotransferase; UST, Uronyl 2-O-sulfotransferase.

The DS-repeating disaccharide region, [iduronic acid (IdoA)–GalNAc]_*n*_, is formed by the epimerization of the C-5 position of GlcA residues in a chondroitin as a precursor molecule (Figure 2A; Maccarana et al., 2006; Pacheco et al., 2009).

**FIGURE 3:**
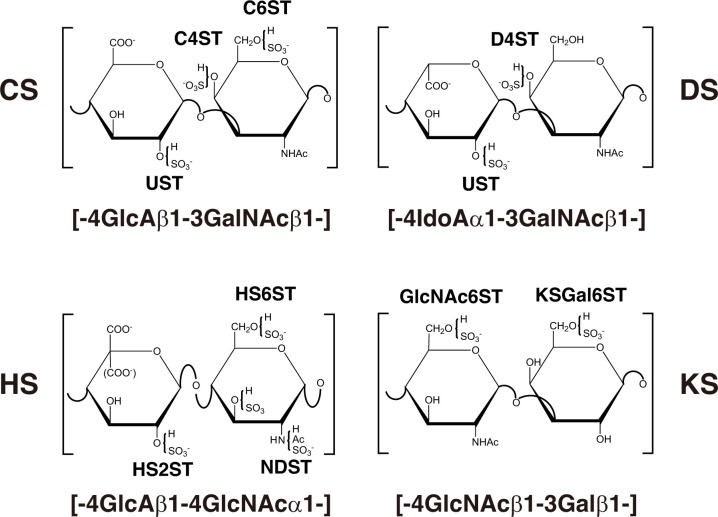
Typical repeating disaccharide units in GAGs and the respective sulfotransferase. The CS backbone consists of GlcA and GalNAc, whereas DS is a stereoisomer of CS that includes IdoA instead of GlcA. HS consist of uronic acids (IdoA and GlcA) and GlcNAc/GlcNH_2_ residues with varying proportions of IdoA and GlcA. These sugar residues can be esterified by sulfate at various positions as indicated in the figure by action of the respective sulfotransferase, which transfers the sulfate group from the sulfate donor 3′-phosphoadenosine 5′-phosphosulfate (PAPS) to the respective hydroxygroup of each residue.

The repeating disaccharide region of HS, [GlcA-GlcNAc]_*n*_, is assembled by exostosin (EXT) family proteins such as EXT1, EXT2, EXT-like 1 (EXTL1), EXTL2, and EXTL3 using UDP-GlcA and UDP-GlcNAc as donor substrates in the Golgi apparatus (Figure 2A and Table 3; Lind et al., 1998; McCormick et al., 1998; Kitagawa et al., 1999; Kim et al., 2001, 2003). After the formation of these polymers, various sulfotransferases transfer a sulfate group to the respective hydroxy and/or amino group in CS, DS, and HS, which gives rise to functional domains to interact with a wide range of proteins (Figure 3 and Tables 2, 3; Kusche-Gullberg and Kjellén, 2003).

**TABLE 3:** Congenital disorders of HS deficiency*.

Gene	Protein	Chromosomal location	MIM number	Name of disorder	Clinical hallmarks
*EXT1*	HS-polymerase-1 (GlcNAcT-II HS-GlcAT-II)	8q24.11	133700 215300 608177	Exostoses multiple type 1; Chondrosarcoma	The formation of cartilage-capped tumors (exostoses) that develop from the growth plate of endochondral bones, particularly long bones.
			150230	Trichorhinophalangeal syndrome, type II; Langer-Giedion syndrome	Craniofacial dysmorphism, exostoses, sparse scalp hair, bushy eyebrows, a bulbous nose, long philtrum, short stature, and cone-shaped epiphyses (This disorder was caused by deletion encompassing *EXT2* and *TRPS1*.)
*EXT2*	HS-polymerase-2 (GlcNAcT-II HS-GlcAT-II)	11p11.2	133701 608210	Exostoses multiple type 2	The formation of cartilage-capped tumors (exostoses) that develop from the growth plate of endochondral bones, particularly long bones.
			616682	Seizures-scoliosis-macrocephaly syndrome	Moderate intellectual disability, seizure disorder, hypotonia, scoliosis, macrocephaly, coarse facies, bilateral cryptorchidism in males, a long hypoplastic philtrum, and hypertelorism.
			601224	Potocki-Shaffer syndrome	Multiple exostoses, bilateral parietal foramina, intellectual disability, hearing loss, craniofacial dysmorphisms, cardiovascular, ocular, and genitourinary tract abnormalities (This disorder was caused by deletion encompassing *EXT2* and eight other genes.)
*EXTL3*	EXTL3 (GlcNAcT-I, GlcNAcT-II)	8p21.1	617425 605744	Immunoskeletal dysplasia with neurodevelopmental abnormalities; Neuro-immuno-skeletal dysplasia syndrome; Spondyloepimetaphyseal dysplasia with immunodeficiency	Severe combined immunodeficiency with a complete absence of T cells, intellectual disability, a short stature, limb shortening, dysmorphic facial features, and skeletal abnormalities such as severe platyspondyly, lumbar gibbus, and kyphoscoliosis.
*NDST1*	NDST-1	5q33.1	616116 600853	Intellectual disability autosomal recessive 46	Impairment in motor and cognitive functions, muscular hypotonia, epilepsy, postnatal growth deficiency, seizures, cranial nerve dysfunction, gastroesophageal reflux, seizure, ataxia, developmental delays, head sparing failure, distinctive facial features, and a bifid uvula.
*HS2ST1*	HS2ST-1	1p22.3	604844 619194	Neurofacioskeletal syndrome with or without renal agenesis	Facial dysmorphism with a coarse face, upslanted palpebral fissures, broad nasal tip, and wide mouth, developmental delay and/or intellectual disability, corpus callosum agenesis or hypoplasia, flexion contractures, brachy- dactyly of hands and feet with broad fingertips and toes, and uni- or bilateral renal agenesis.
*HS6ST1*	HS6ST-1	2q14.3	614880 604846	Hypogonadotropic hypogonadism 15 with or without anosmia; Kallmann syndrome	Lack of sexual maturation, low levels of circulating gonadotropins and testosterone, and anosmia.
*SULF1*	HS 6-*O*-endosulfatase-1	8q13.2-q13.3	610012	Mesomelia-synostoses syndrome	Mesomelic limb shortening, acral synostoses, and multiple congenital malformations (This disorder was caused by deletion 582-738 kb in size encompassing *SULF1* and *SLCO5A*1.)

*, the table was cited from a reference (Mizumoto, 2018) with modifications. EXT, exostosin; EXTL, exostosin-like; NDST, N-deacetylase/N-sulfotransferase; HS2ST, heparan sulfate 2-O-sulfotransferase; HS6ST, heparan sulfate 6-O-sulfotransferase.

The repeating disaccharide region of KS, [Gal-GlcNAc]_*n*_, is assembled by GalT and GlcNAcT encoded by *B4GALT4* and *B3GNT7*, respectively (Figure 2B; Seko et al., 2003; Seko and Yamashita, 2004; Kitayama et al., 2007). Further modification by sulfation on Gal and GlcNAc residues occurs by the action of KS Gal 6-*O*-sulfotransferase and GlcNAc 6-*O*-sulfotransferase encoded by *CHST1* and *CHST5* as well as *CHST6*, respectively (Figure 3 and Table 4; Fukuta et al., 1997; Akama et al., 2001, 2002; Narentuya et al., 2019).

**TABLE 4:** Congenital disorders of KS deficiency.

Gene	Protein	Chromosomal location	MIM number	Name of disorder	Clinical hallmarks
*CHST6*	Corneal GlcNAc 6-*O*-sulfotransferase	16q23.1	605294 217800	Macular corneal dystrophy	Progressive punctate opacities in the cornea, bilateral loss of vision.

## Proteoglycan Linkeropathy

The disturbance of construction of the linker region tetrasaccharide, -*O*-Xyl-Gal-Gal-GlcA, in CS, DS, and HS by mutations in *XYLT1*, *XYLT2*, *B4GALT7*, *B3GALT6*, and *B3GAT3* causes the hereditary disease “Proteoglycan linkeropathy,” which is characterized by abnormalities in connective tissue, bone, skin, and heart (Table 1). Proteoglycan linkeropathy, designated by Dr. Shiro IKEGAWA, is a collective term for diverse connective tissue disorders caused by mutations in these genes (Nakajima et al., 2013). Nosology and classification of skeletal disorders and Ehlers-Danlos syndrome (EDS) are referred to in the Nosology and classification of genetic skeletal disorders: 2019 revision and 2017 International Classification of the Ehlers–Danlos syndromes, respectively (Malfait et al., 2017, 2020; Mortier et al., 2019).

### Disorders Caused by Mutations of *XYLT1* and *XYLT2*

XYLT1 transfers a Xyl residue from UDP-Xyl to the serine residue on the specific core proteins of PGs (Figure 2A; Götting et al., 2000). Desbuquois dysplasia type 2 is an autosomal recessive disorder that is characterized by severe pre- and postnatal growth retardation, a short stature, joint laxity, the dislocation of large joints, and a flat face with prominent eyes (Table 1; Desbuquois et al., 1966), and is caused by mutations in *XYLT1* (Bui et al., 2014; Silveira et al., 2016; Al-Jezawi et al., 2017; Guo et al., 2017a). Fibroblasts from patients (p.Arg147^∗^) show reduced CS rather than HS (Bui et al., 2014), which may indicate that the loss-of-function in XYLT1 affects the biosynthesis of CS rather than HS.

Autosomal recessive short stature syndrome was also caused by a homozygous mutation (p.Arg481Trp) in *XYLT1* (Schreml et al., 2014). Although XYLT1 in control cells was located in the Golgi apparatus, p.Arg481Trp-XYLT1 was diffusely distributed throughout the cytoplasm with partial localization to the Golgi apparatus in patients’ fibroblasts (Schreml et al., 2014). Furthermore, decorin-PG from the fibroblasts of a patient was partially deglycanated (Schreml et al., 2014).

Baratela-Scott syndrome is an autosomal recessive disorder characterized by a short stature, patellar dislocation, short tubular bones, mild metaphyseal changes, brachymetacarpalia with stub thumbs, short femoral necks, shallow acetabular roofs, platyspondyly, a flattened midface with broad nasal bridge, cleft palate, bifid uvula, and synophrys (Baratela et al., 2012). This disease is caused by homozygous mutations in *XYLT1*, homozygous hypermethylation of exon 1 in *XYLT1* that is caused by a GGC repeat expansion in the XYLT1 promoter region, or a heterozygous 3.1 Mbp deletion on 16p13 encompassing XYLT1 with hypermethylation (LaCroix et al., 2019).

Homozygous mutations in *XYLT2* cause spondyloocular syndrome that is characterized by retinal detachment, amblyopia, nystagmus, hearing loss, heart septal defects, bone fragility, and mild learning difficulties (Table 1; Munns et al., 2015; Taylan et al., 2016, 2017; Umair et al., 2018). The levels of GAGs and XYLT activity in the dermal fibroblasts of a patient were lower than those of healthy subjects (Munns et al., 2015).

The different clinical manifestations between Desbuquois skeletal dysplasia type 2 and spondyloocular syndrome may indicate that XYLT1 and XYLT2 do not compensate for each other, and that the serine residue(s) of the respective core protein as a substrate for each XYLT and spatiotemporal expression may be distinct between XYLT1 and XYLT2.

### Severe (Lethal) Neonatal Short Limb Dysplasia With Multiple Dislocations Caused by Mutations in *FAM20B*

*FAM20B* encodes a kinase, which phosphorylates a Xyl residue in the linker region tetrasaccharide of GAGs (Koike et al., 2009). Compound heterozygous mutations (p.Thr59Alafs^∗^19/p.Asn347Metfs^∗^4) in *FAM20B* cause neonatal short limb dysplasia resembling Desbuquois dysplasia (Table 1; Kuroda et al., 2019). The clinical hallmarks are mid-face hypoplasia, thoracic hypoplasia with respiratory failure, a very short stature with mesomelic shortening of the limbs, multiple dislocations of large joints, and preaxial digital hypoplasia as well as syndactyly, being similar to Desbuquois dysplasia (Kuroda et al., 2019).

### Ehlers-Danlos Syndrome Spondylodysplastic Type 1 and Larsen Syndrome Reunion Island Type Caused by Mutations in *B4GALT7*

*B4GALT7* encodes GalT-I, which transfers the second Gal residue in the linker region tetrasaccharide from UDP-Gal to the serine-*O*-Xyl (Figure 2A; Almeida et al., 1999; Okajima et al., 1999). Compound heterozygous and homozygous mutations in *B4GALT7* cause Ehlers-Danlos syndrome (EDS) spondylodysplastic type 1 (Table 1; Almeida et al., 1999; Okajima et al., 1999; Malfait et al., 2017, 2020). Patients show various clinical symptoms including an aged appearance, craniofacial dysmorphism, hypermobile joints, generalized osteopenia, a short stature, hypotonic muscles, defective wound healing, global developmental delays, and scoliosis (Almeida et al., 1999; Guo et al., 2013; Arunrut et al., 2016; Salter et al., 2016; Ritelli et al., 2017; Mihalic Mosher et al., 2019). Skin fibroblasts of patients were shown to be defective for the DS side chain of DS-PGs, decorin and biglycan, and show a marked reduction in GalT-I activity *in vitro* (Quentin et al., 1990; Seidler et al., 2006). Furthermore, fibroblasts from patients exhibited reduced levels of sulfation on HS chains (Götte et al., 2008). Hence, various clinical manifestations in EDS spondylodysplastic type 1 might partially result from defects in not only DS, but also HS. Namely, the reasons why these patients with the same *B4GALT7* gene mutations exhibited the wide range of symptoms might be due to the distinct mutational positions in *B4GALT7*, resulting in the different influence of enzymatic activity and/or intracellular localization of mutant enzymes and in the immature GAGs produced by the mutant enzymes.

Larsen syndrome Reunion Island type is caused by a homozygous mutation in *B4GALT7* (Table 1; Cartault et al., 2015). The characteristic symptoms of Larsen syndrome are congenital large-joint dislocations including the hip, elbow, and knee, craniofacial abnormalities, and foot deformities (Larsen et al., 1950). Patients showed similar hallmarks to EDS spondylodysplastic type 1 caused by a mutation in *B4GALT7*, including dwarfism, distinctive facial features, and hyperlaxity, in addition to the classical symptoms of Larsen syndrome (Cartault et al., 2015). Thus, EDS spondylodysplastic type 1 and Larsen syndrome Reunion Island type may share clinical spectra.

### Ehlers-Danlos Syndrome Spondylodysplastic Type 2 and Spondyloepimetaphysial Dysplasia With Joint Laxity Type 1 Caused by Mutations in *B3GALT6*

*B3GALT6* encodes GalT-II, which transfers the third Gal residue in the linker region tetrasaccharide from UDP-Gal to serine-*O*-Xyl-Gal (Figure 2A; Bai et al., 2001). Compound heterozygous mutations in *B3GALT6* cause EDS spondylodysplastic type 2, which is characterized by kyphoscoliosis, clubfeet, elbow malalignment, muscle hypotonia, wrinkled skin, and a characteristic facial appearance (Table 1; Malfait et al., 2013, 2020; Nakajima et al., 2013). Skin fibroblast cultures from patients produced less CS, DS, and HS than those from control cells (Malfait et al., 2013).

Spondyloepimetaphyseal dysplasia with joint laxity type 1 is also caused by compound heterozygous mutations in *B3GALT6* (Table 1; Nakajima et al., 2013; Ritelli et al., 2014; Vorster et al., 2015; Alazami et al., 2016). Spondyloepimetaphyseal dysplasia with joint laxity type 1 is characterized by kyphoscoliosis, joint laxity, hip dislocation, elbow contracture, platyspondyly, clubfeet, and craniofacial dysmorphisms (Beighton et al., 1984). The recombinant mutant enzymes showed a significantly lower GalT-II activity than wild-type B3GALT6 (Nakajima et al., 2013). Furthermore, the production of HS and CS/DS in cultured lymphoblastoid cells from patients was shown to be lower and higher, respectively, than that in normal cells (Nakajima et al., 2013). In addition, the levels of HS and HS-PG, perlecan, were markedly lower in cultured fibroblasts from patients than those from normal subjects, whereas the levels of CS-PG and DS-PG, versican and decorin, respectively, in the fibroblasts of patients corresponded to the fibroblasts of normal subjects (Ritelli et al., 2014). These findings suggest that the causative mutations of spondyloepimetaphyseal dysplasia with joint laxity type 1 may affect the biosynthesis of only HS, and not CS or DS, which results in the skeletal and joint hallmarks of both disorders.

### Disorders Caused From *B3GAT3* Mutations

*B3GAT3* encodes GlcAT-I, which transfers the 4th GlcA residue in the linker region tetrasaccharide, Xyl-Gal-Gal-GlcA, from UDP-GlcA to the serine-*O*-Xyl-Gal-Gal (Figure 2A; Kitagawa et al., 1998). Homozygous mutations in *B3GAT3* cause multiple joint dislocations, a short stature, kyphoscoliosis, craniofacial dysmorphism, and heart defects including bicuspid aortic valve and aortic root dilatation (Table 1; Baasanjav et al., 2011; von Oettingen et al., 2014; Budde et al., 2015). GlcAT-I activity and the levels of CS, DS, and HS in fibroblasts from these patients were significantly lower than those in the fibroblasts of control cells (Baasanjav et al., 2011; Budde et al., 2015). Other homozygous mutations in *B3GAT3* caused distinct hallmarks such as a short stature, spondyloepimetaphyseal dysplasia, osteoporosis, fractures, multiple bony chondromas, cutis laxa, blue sclerae, glaucoma, bilateral radio-ulnar synostosis, severe osteopenia, bilateral clubfeet, atrial and ventricular septal defects, hypertelorism, a small chest, diaphragmatic hernia, arachnodactyly, hypotonia, hearing loss, and perinatal cerebral infarction with bilateral supra- and infratentorial subdural hematomas (Jones et al., 2015; Alazami et al., 2016). Moreover, a patient with the compound heterozygous mutation in *B3GAT3* exhibited severe osteoporosis, fractures, scoliosis, joint laxity, hypotonia, a bicuspid aortic valve, and myopia (Job et al., 2016).

Compound heterozygous mutation in *B3GAT3* causes pseudodiastrophic dysplasia, which is associated with prenatal manifestation and early lethality (Table 1; Byrne et al., 2020). Pseudodiastrophic dysplasia is characterized by a short stature at birth, facial dysmorphism, and distinctive skeletal abnormalities including short ribs, platyspondyly, broad ilia with flaring, an increased acetabular angle, shortened long bones with metaphyseal flaring, elongation of the proximal and middle phalanges with subluxation of the proximal interphalangeal joints, subluxation of the elbow, and talipes equinovarus (Eteson et al., 1986; Fischetto et al., 1997; Yap et al., 2016). GlcAT-I activity of fibroblasts from a patient was markedly lower than that from healthy control cells (Byrne et al., 2020). Accordingly, the levels of CS, DS, and HS from the patient cells were significantly decreased compared with control cells (Byrne et al., 2020). Thus, this mutation, p.Arg169Trp/Arg225X, may be a complete loss of function of B3GAT3, which is responsible for the most severe and lethal manifestation of skeletal dysplasia.

Yauy et al. (2018) proposed the classification of *B3GAT3*-related disorders based on the diverse symptoms of patients with distinct mutations in *B3GAT3* (Table 1): (1) “B3GAT3-related disorder with joint dislocation and congenital heart defect,” which is similar to Larsen syndrome; (2) “B3GAT3-related disorder with craniosynostosis and bone fragility,” which is similar to Antley-Bixler and Shprintzen-Goldberg syndromes; (3) “B3GAT3-related disorder with cutis laxa and bone fragility,” which is similar to geroderma osteodysplastica syndrome; and (4) “B3GAT3-related disorder with intermediate phenotype,” which is similar to Larsen syndrome and Antley-Bixler/Shprintzen-Goldberg syndromes (Yauy et al., 2018). Patients with a broad spectrum of hallmarks caused by mutations in the same *B3GAT3* may show various degree of affected biosynthesis of CS, DS, and HS, which is consistent with other linkeropathies. Further analyses using cells from patients with the four types of mutations in *B3GAT3* are required to understand the underlying pathogenic mechanisms.

## Congenital Disorders of Chondroitin Sulfate Deficiency

### Mild Skeletal Dysplasia With Joint Laxity and Advanced Bone Age by Mutations in *CSGALNACT1*

*CSGALNACT1* encodes *N*-acetylgalactosaminyltransferase-I (GalNAcT-I) and GalNAcT-II, which transfer the 5th GalNAc and subsequent GalNAc residues (7, 9, 11th- - -) from UDP-GalNAc to serine-*O*-Xyl-Gal-Gal-GlcA and [GalNAc-GlcA]_*n*_, respectively (Figure 2A; Uyama et al., 2002, 2003). Homozygous or compound heterozygous mutations in *CSGALNACT1* cause mild skeletal dysplasia, joint laxity, a short stature with an advanced bone age, facial dysmorphism, and mild language delay (Table 2; Vodopiutz et al., 2017; Mizumoto et al., 2020). GalNAcT activity was significantly reduced in fibroblasts from patients. In addition, the amount of CS was decreased in patients’ fibroblasts compared with control fibroblasts (Mizumoto et al., 2020).

### Temtamy Preaxial Brachydactyly Syndrome Caused by Mutations in *CHSY1*

*CHSY1* encodes a dual glycosyltransferase, which functions as both GalNAcT-II and CS-GlcAT-II, transferring GalNAc and GlcA residues from UDP-GalNAc and UDP-GlcA, respectively (Figure 2A; Kitagawa et al., 2001, 2003; Izumikawa et al., 2007, 2008). Homozygous or compound heterozygous mutations in *CHSY1* cause Temtamy preaxial brachydactyly syndrome, which is characterized by preaxial brachydactyly, hyperphalangism of digits, facial dysmorphism, dental anomalies, sensorineural hearing loss, delayed motor and mental development, and growth retardation (Table 2; Temtamy et al., 1998, 2012; Li et al., 2010; Tian et al., 2010; Sher and Naeem, 2014). The level of CS in a skin section from a patient was lower than that in control skin (Tian et al., 2010). Furthermore, the up-regulated expressions of *JAG1* and *HES1*, whose gene products are ligands of NOTCH and induced by NOTCH signaling, respectively, have been observed (Tian et al., 2010). NOTCH signaling is involved in appendicular skeletal development (Tian et al., 2010). Therefore, a decrease in CS by mutations in CHSY1 may influence changes in the expression of *JAG1* and *HES1*, thereby leading to brachydactyly.

### Spondyloepiphyseal Dysplasia With Congenital Joint Dislocations, Larsen Syndrome, and Humero-Spinal Dysostosis Caused by Mutations in *CHST3*

*CHST3* encodes chondroitin 6-*O*-sulfotransferase 1 (C6ST1), which transfers a sulfate group from 3′-phosphoadenosine 5′-phosphosulfate (PAPS) to the C-6 hydroxy group of GalNAc residues in CS chains (Figure 3; Fukuta et al., 1998). Homozygous mutations in *CHST3* cause spondyloepiphyseal dysplasia with congenital joint dislocations, which is characterized by a short stature, severe kyphoscoliosis, mild brachydactyly, arthritic joints, joint dislocation, rhizomelia, fusion of the carpal bones, metacarpal shortening, osteoarthritis of the elbow, deafness, and ventricular septal, mitral, and/or tricuspid defects (Table 2; Rajab et al., 2004; Thiele et al., 2004; van Roij et al., 2008; Tuysuz et al., 2009; Tanteles et al., 2013; Muys et al., 2016; Waryah et al., 2016; Srivastava et al., 2017). Loss of C6ST activity as well as the decrease of 6-*O*-sulfated CS were demonstrated in skin fibroblasts from patients (Thiele et al., 2004; van Roij et al., 2008). These findings suggest that chondroitin 4-*O*-sulfotransferase (C4ST) and/or 4-*O*-sulfation of CS cannot compensate for the loss-of-function in C6ST1.

Homozygous or heterozygous mutations in *CHST3* cause Larsen syndrome and humero-spinal dysostosis, which are characterized by joint and knee dislocations, bilateral clubfeet, and elbow joint and spinal dysplasias (Table 2; Hermanns et al., 2008). Although there are a wide range of diagnoses for multiple dislocations in a newborn, Larsen syndrome is one of the most common entities (Hermanns et al., 2008). Hence, the different pathological diagnoses by mutations in *CHST3* may be due to age-related descriptions of the same conditions (Hermanns et al., 2008). Twenty-four patients from 23 families with mutations in *CHST3* were diagnosed with Larsen syndrome (15 families), humero-spinal dysostosis (four cases), chondrodysplasia with multiple dislocations (Megarbane type; two cases), Desbuquois syndrome (one case), and spondyloepiphyseal dysplasia (one case) (Unger et al., 2010).

### Osteochondrodysplasia, Brachydactyly, and Overlapping Malformed Digits Caused by Mutations in *CHST11*

*CHST11* encodes chondroitin 4-*O*-sulfotransferase 1 (C4ST1), which transfers a sulfate group from PAPS to the C-4 hydroxy group of GalNAc residues in CS chains (Figure 3; Hiraoka et al., 2000; Yamauchi et al., 2000). Homozygous in-frame deletion of 15 nucleotides in *CHST11*, which results in the deletion of amino acids (Lys156-Asn160), causes limb malformations including brachydactyly, overriding digits, clino-symphalangism, syndactyly, and hexadactyly, and skeletal defects including scoliosis, dislocation of patellae and fibulae, and pectus excavatum (Table 2; Shabbir et al., 2018). Biochemical analyses using a recombinant enzyme as well as patients’ cells have not been conducted to our knowledge.

The deletion of a 55-kb region within chromosome 12q23 that encompasses a part of *CHST11* and an embedded microRNA, MIR3922, causes defects of the digits of the upper and lower limbs and malignant lymphoproliferative disease (Chopra et al., 2015). Although the digital malformations are consistent with the hallmarks of the proband who was homozygous for an in-frame deletion of 15 nucleotides in *CHST11*, as described above, the lymphoproliferative disorder is not. Hence, it remains unclear whether the deletion in *CHST11* contributes to T-cell lymphoproliferative disorder. It should be noted that compound heterozygous variants in *MBD3L5* and *PRND* and homozygous missense variants in *EPHA10* and *OLA1* were also identified in the proband (Chopra et al., 2015). Further analysis of CHST11 and/or 4-*O*-sulfation of CS in the differentiation as well as proliferation of lymphocytes is required.

## Congenital Disorders of Dermatan Sulfate Deficiency

### Ehlers-Danlos Syndrome Musculocontractural Type 2 Caused by Mutations in *DSE*

*DSE* encodes dermatan sulfate epimerase (DSE), which converts GlcA into IdoA by epimerizing the C-5 carboxy group of GlcA residues in the repeating disaccharide region of the chondroitin precursor chain, [GlcA-GalNAc]_*n*_, to form the disaccharide region of DS, [IdoA-GalNAc]_*n*_ (Figure 2A; Maccarana et al., 2006). The homozygous mutations in *DSE* cause EDS musculocontractural type 2 (Table 2; Müller et al., 2013; Syx et al., 2015; Lautrup et al., 2020; Malfait et al., 2020). The clinical hallmarks of the patients exhibited characteristic facial features including hypertelorism, blue sclera, midfacial hypoplasia, contracture of the thumbs and feet, atrophic scars on the skin, and hypermobility of the finger, elbow, and knee joints (Müller et al., 2013; Syx et al., 2015). DSE activity of the fibroblasts from the patient was markedly decreased compared with healthy control cells, which resulted in a lower level of DS, being accompanied by an increase in the level of CS (Müller et al., 2013). These findings indicate that mutations in *DSE* may cause a decrease in DS, leading to EDS musculocontractural type 2. In order to elucidate the underlying pathogenic mechanism of EDS, it will be necessary to analyze the signaling pathway(s) regulated by DS as well as functional protein(s) interacting with DS chains of DS-PGs.

### Bipolar Disorder and Diaphragmatic Hernia Caused by Mutations in *DSEL*

*DSEL* encodes dermatan sulfate epimerase-like (DSEL)/DSE2, which is a homolog of DSE and can catalyze the reaction converting GlcA into IdoA (Pacheco et al., 2009). The 113 probands with bipolar disorder characterized by alternating episodes of depression were identified as caused by various homozygous as well as heterozygous mutations in *DSEL*, such as the substitution of adenine to guanine in the 5′-non-coding region 546 bp upstream of the coding region, p.Val287Ile, p.Pro673Ser, p.Tyr730Cys, p.Pro942Ser, and p.Ile1113Met, in *DSEL* (Table 2; Goossens et al., 2003). Moreover, the recurrent early-onset major depressive disorder was caused by single nucleotide polymorphism (SNP), rs17077540, which was detected 75 kbp upstream of *DSEL* (Shi et al., 2011). DSEL but not DSE was demonstrated to be expressed predominantly in the brain (Table 2; Nakao et al., 2000; Maccarana et al., 2006; Akatsu et al., 2011; Bartolini et al., 2012). Functional analyses of DSEL using neuronal cells and model organisms such as the mouse and zebrafish, may lead to elucidation of the pathogenic mechanism of this disorder.

The deletion of 2.7 Mbp at chromosome 18q22.1, which contains three genes, *CDH19*, *DSEL*, and *TXNDC10/TMX3* encoding cadherin 19, DSEL/DSE2, and thioredoxin domain-containing protein10/thioredoxin related transmembrane protein 3, respectively, causes diaphragmatic hernia (Zayed et al., 2010). Furthermore, the heterozygous substitution, c.1515G > A (p.Met14Ile), in *DSEL* also results in the disease (Table 2; Zayed et al., 2010). Thus, DSEL and/or DS might contribute to the development of the diaphragm, although the possibility of the involvement of CDH19 and TXNDC10/TMX3 in this disorder cannot be excluded.

### Ehlers-Danlos Syndrome Musculocontractural Type 1 Caused by Mutations in *CHST14*

*CHST14* encodes dermatan 4-*O*-sulfotransferase 1 (D4ST1), which transfers a sulfate group from PAPS to the C-4 hydroxy group of GalNAc residues in DS chains (Figure 3; Evers et al., 2001; Mikami et al., 2003). Homozygous, complex homozygous, or compound heterozygous mutations in *CHST14* cause EDS musculocontractural type 1 (Table 2; Dündar et al., 2009; Malfait et al., 2010, 2020; Miyake et al., 2010), which is characterized by kyphoscoliosis, muscular hypotonia, hyperextensible, thin, and bruisable skin, atrophic scarring, joint hypermobility, multiple joint contracture, characteristic craniofacial features, joint laxities, and recurrent dislocations (Kosho et al., 2005, 2010; Dündar et al., 2009; Malfait et al., 2010, 2020; Miyake et al., 2010). Fibroblasts from probands with the mutations in CHST14 showed markedly lower D4ST activity than that from the respective control (Miyake et al., 2010). Surprisingly, CS, instead of non-sulfated DS (dermatan), was produced on the decorin core protein from skin fibroblasts of probands (Miyake et al., 2010). Collagen fibrils were dispersed in the patient dermis; in contrast, they were regularly and tightly assembled in the healthy controls (Hirose et al., 2019). The affected GAG side chain on decorin was altered from DS to CS. Since CS has a linear conformation, the CS side chain on decorin stretched from the outer surface of collagen fibrils to adjacent fibrils (Hirose et al., 2019). In contrast, DS has a curved conformation, and the DS side chain on decorin maintained close contact with attached collagen fibrils. Therefore, alteration of the side chain on decorin from DS to CS caused by loss-of-function in *CHST11* results in the spatial disorganization of collagen networks, which may disrupt the appropriate structure of GAG side chains surrounding collagen fibrils (Hirose et al., 2019). Functional analyses of *CHST14* using knockout mice are ongoing (Yoshizawa et al., 2018; Hirose et al., 2021; Nitahara-Kasahara et al., submitted).

Homozygous mutations or compound heterozygous mutation in *CHST14* caused adducted thumb-clubfoot syndrome in 11 patients from 4 families (Table 2; Dündar et al., 2009). This syndrome is characterized by congenital contracture of the thumbs and feet, joint instability, a typical facial appearance, facial clefting, coagulopathy, thin and translucent skin, connective tissue fragility with aging, and defects in the heart, kidneys, or intestines (Dundar et al., 1997; Sonoda and Kouno, 2000; Janecke et al., 2001). Five out of eleven probands with adducted thumb-clubfoot syndrome died in early infancy or childhood (Dundar et al., 1997). Malfait et al. (2010) proposed to unify EDS and the adducted thumb-clubfoot syndrome, caused by mutations in *CHST14*, as “musculocontractural EDS,” which was adopted in the 2017 International Classification of the Ehlers–Danlos syndromes as “EDS musculocontractural type 1” (Table 2; Malfait et al., 2017).

## Congenital Disorders of Heparan Sulfate Deficiency

### Multiple Osteochondromas Caused by *EXT1* or *EXT2* Mutations

*EXT1* encodes exostosin 1 (EXT1), a dual glycosyltransferase (GlcNAcT-II and HS-GlcAT-II), which transfers the GlcNAc and GlcA residues from UDP-GlcNAc and UDP-GlcA, respectively, to [GlcA-GlcNAc]_*n*_ (Figure 2A; Lind et al., 1998; McCormick et al., 1998). *EXT2* also encodes a dual glycosyltransferase, which has activities of both GlcNAcT-II and HS-GlcAT-II (Lind et al., 1998; McCormick et al., 1998). The hetero-dimeric complex of EXT1 and EXT2 shows polymerization activity to construct repeating disaccharides in HS, [GlcA-GlcNAc]_*n*_, by alternating addition of GlcA and GlcNAc from UDP-GlcA and UDP-GlcNAc, respectively, *in vitro* (Figure 2A; Kim et al., 2003). Heterozygous mutations in *EXT1* or *EXT2* cause autosomal dominant disorder, osteochondroma, which is characterized by cartilaginous capped bony outgrowths located at the growth plates of long bones, a short stature, camptomelic dysplasia including the forearm and crus, early-onset osteoarthritis, and malignant transformation in approximately 1% of patients (Table 3; Solomon, 1963; Hennekam, 1991; Ahn et al., 1995; Stickens et al., 1996; Philippe et al., 1997; Wuyts et al., 1998; Bovée et al., 1999; Wuyts and Van Hul, 2000; Cheung et al., 2001; Faiyaz-Ul-Haque et al., 2004). To our knowledge, 436 and 223 mutations in *EXT1* and *EXT2*, respectively, have been identified to date.

Langer-Giedion syndrome (trichorhinophalangeal syndrome type II) is an autosomal dominant disorder caused by a deletion in chromosome 8q23.3-q24.11, which includes *EXT1* and *TRPS1* encoding exostosin 1 and transcriptional repressor GATA binding 1, respectively (Table 3; Lüdecke et al., 1995). Langer-Giedion syndrome is characterized by multiple osteochondromas, cone-shaped epiphyses, a short stature, bulbous nose, sparse scalp hair, a long philtrum, and bushy eyebrows (Lüdecke et al., 1995). A deletion in chromosome 8q24.11 or 8q23.3-q24.13, which contains *EXT1*, but not *TRPS1*, also causes Langer-Giedion syndrome (Wuyts et al., 2002; McBrien et al., 2008; Pereza et al., 2012).

A heterozygous deletion of 2.3 Mbp in chromosome 11p11.21 containing *EXT2* causes Potocki-Shaffer-syndrome that is characterized by multiple osteochondromas, developmental delays, biparietal foramina, craniofacial abnormalities, and intellectual disability (Table 3; Swarr et al., 2010; Palka et al., 2012). These hallmarks, except for multiple osteochondromas, may be caused by the lack of eight other genes in the chromosome 11p11.21.

### Seizures-Scoliosis-Macrocephaly Syndrome Caused by Mutations in *EXT2*

The complex homozygous mutations in *EXT2* cause seizures-scoliosis-macrocephaly syndrome, but not multiple osteochondromas (Table 3; Farhan et al., 2015). The seizures-scoliosis-macrocephaly syndrome is characterized by seizures, intellectual disability, hypotonia, scoliosis, macrocephaly, hypertelorism, and renal dysfunction (Farhan et al., 2015). The protein level of the mutant EXT2, p.Met87Arg/p.Arg95Cys, in fibroblasts from patients was lower than that from control fibroblasts (Farhan et al., 2015). These findings indicate that EXT2 and/or HS side chains of HS-PGs may contribute to normal brain development, possibly by regulating the signaling pathway of Wnt as well as fibroblast growth factor (FGF) and/or by controlling the assembly of extracellular matrix. Wnt and FGF signalings were shown to be affected during nervous system development in zebrafish by mutants in *ext2/dackel* (Lee et al., 2004; Fischer et al., 2011).

### Immunoskeletal Dysplasia With Neurodevelopmental Abnormalities Caused by Mutations in *EXTL3*

*EXTL3* encodes exostosin-like-3 (EXTL3), a dual glycosyltransferase (GlcNAcT-I and GlcNAcT-II), which transfers a GlcNAc residue from UDP-GlcNAc to the GlcA residue at the non-reducing end of the common GAG-linker region tetrasaccharide and to the GlcA residue in the repeating disaccharide region of HS chains, respectively (Figure 2A; Kim et al., 2001). The homozygous mutations in *EXTL3* cause immunoskeletal dysplasia with neurodevelopmental abnormalities that is characterized by severe platyspondyly, kyphoscoliosis, pelvic distortion, constriction of the proximal femora and brachydactyly, neurological abnormalities including generalized seizures, opisthotonus, hyperreflexia, intellectual disability, nystagmus, muscular hypotonia, and a decrease in T-lymphocyte subsets as well as immunoglobulins (Table 3; Guo et al., 2017b; Oud et al., 2017; Volpi et al., 2017). Although wild-type EXTL3 from a healthy subject localized to the Golgi apparatus, p.Arg513Cys-EXTL3 from a patient did not (Oud et al., 2017). Therefore, the mislocalization of the mutant EXTL3 (p.Arg513Cys) may cause loss-of-function of EXTL3. Moreover, HS levels in the fibroblasts of patients were markedly lower than those in healthy controls (Oud et al., 2017). Up-regulation of FGF signaling as well as increase in the 2-*O*- and 6-*O*-sulfation of HS were observed in the fibroblasts of a patient with a homozygous mutation in *EXTL3* (Volpi et al., 2017), being consistent with the reports that FGF signaling requires both 2-*O*- and 6-*O*-sulfation on HS. The reduced expansion of lymphohematopoietic progenitor cells and defects in the differentiation of thymic epithelial progenitor cells were also detected in the induced pluripotent stem (iPS) cells of the patient (Volpi et al., 2017). These findings suggest that EXTL3 and/or HS-PGs play roles in not only skeletal development, but also cell differentiation of the nervous and immune systems. Their functions in specific organs such as the brain and thymus are under investigation using conditional knockout mice.

### Intellectual Disability Due to Autosomal Recessive State Caused by Mutations in *NDST1*

*NDST1* encodes GlcNAc *N*-deacetylase/*N*-sulfotransferase 1 (NDST1), which has bifunctional domains including an *N*-deacetylase and *N*-sulfotransferase, acting on GlcNAc residues in HS chains (Figure 3; Hashimoto et al., 1992; Dixon et al., 1995). The homozygous mutations in *NDST1* cause autosomal recessive intellectual disability, muscular hypotonia, epilepsy, and postnatal growth deficiency (Table 3; Najmabadi et al., 2011; Reuter et al., 2014). Furthermore, the compound heterozygous mutation in *NDST1* causes developmental delay, a short stature, seizures, cranial nerve palsies, ataxia, severe respiratory difficulties in infancy, and distinctive facial features including a long nose and bifid uvula (Table 3; Armstrong et al., 2017). These findings suggest that NDST1 and/or *N*-sulfation in HS chains may contribute to development of the brain and lungs and maintenance of their functions.

### Neurofacioskeletal Syndrome With or Without Renal Agenesis Caused by Mutations in *HS2ST1*

*HS2ST1* encodes HS 2-*O*-sulfotransferase 1 (HS2ST1), which transfers a sulfate group from PAPS to the C-2 hydroxy group of GlcA as well as IdoA residues in HS chains (Figure 3; Kobayashi et al., 1997). Complex heterozygous mutations in *HS2ST1* cause the brachydactyly of hands and feet, flexion contractures, corpus callosum agenesis or hypoplasia, intellectual disability, uni- or bilateral renal agenesis, developmental delay, and facial dysmorphism such as a coarse face, upslanted palpebral fissures, a broad nasal tip, and wide mouth (Table 3; Schneeberger et al., 2020). HS chains of skin fibroblasts from patients lacked 2-*O*-sulfation of GlcA/IdoA residues, resulting in an increase in *N*- and 6-*O*-sulfation of glucosamine residue (Schneeberger et al., 2020). Furthermore, decreased activation of mitogen-activated protein kinase, ERK1/2, by FGF2, was detected in the cell cultures of affected individuals (Schneeberger et al., 2020). These findings indicate that HS2ST1 and 2-*O*-sulfated HS are required for skeletal and renal development in humans.

### Hypogonadotropic Hypogonadism With or Without Anosmia and Kallmann Syndrome Caused by Mutations in *HS6ST1*

*HS6ST1* encodes HS 6-*O*-sulfotransferase 1 (HS6ST1), which transfers a sulfate group from PAPS to the C-6 hydroxy group of GlcNAc and glucosamine residues in HS chains (Figure 3; Habuchi et al., 1998, 2000). The homozygous or heterozygous mutations cause Kallmann syndrome and idiopathic hypogonadotrophic hypogonadism (Table 3; Tornberg et al., 2011). Congenital hypogonadotrophic hypogonadism is characterized by the impaired production or action of gonadotropin-releasing hormone (GnRH), which results in dysfunction of the hypothalamic-pituitary-gonadal hormone axis, leading to low testosterone levels and impaired fertility (Boehm et al., 2015; Millar et al., 2021). Furthermore, congenital hypogonadotrophic hypogonadism is a GnRH deficiency that is associated with other developmental anomalies including a cleft lip or palate, dental agenesis, ear anomalies, hearing impairment, renal agenesis, bimanual synkinesis, and skeletal anomalies (Boehm et al., 2015). Kallmann syndrome is characterized by anosmia or hyposmia in addition to the manifestations of hypogonadotrophic hypogonadism, which results from abnormal embryonic migration of GnRH neurons from their origin in the olfactory placode to the forebrain (Boehm et al., 2015). Congenital hypogonadotrophic hypogonadism and Kallmann syndrome are caused by mutations in *KAL1/ANOS1*, *FGF8*, *FGF17*, and *FGF receptor 1* (*FGFR1*) (Bick et al., 1992; Dodé et al., 2003; Falardeau et al., 2008; Miraoui et al., 2013). Anosmin-1, an extracellular matrix protein, encoded by *KAL1/ANOS1* enhances FGF signaling through interaction with the FGF-FGFR-HS-PG complex on the cell surface (González-Martínez et al., 2004). These findings suggest that 6-*O*-sulfation of GlcNAc and/or glucosamine residues in HS chains is required for their interaction with anosmin-1 and/or FGF, thereby allowing the regulation of FGF signaling.

## Congenital Disorders of Keratan Sulfate Deficiency

### Macular Corneal Dystrophy Caused by Mutations in *CHST6*

*CHST6* encodes a corneal GlcNAc 6-*O*-sulfotransferase (C-GlcNAc6ST), which transfers a sulfate group from PAPS to the C-6 hydroxy group of GlcNAc in KS chains (Figure 3; Akama et al., 2001, 2002). Homozygous, heterozygous, or compound heterozygous mutations in *CHST6* cause macular corneal dystrophy characterized by an autosomal recessive hereditary disease, progressive punctate opacities in the cornea, bilateral loss of vision (Table 4; Akama et al., 2000; El-Ashry et al., 2002; Aldave et al., 2004; Sultana et al., 2005). However, the influence of a defect in *CHST6* on KS biosynthesis remains unclear. Hence, further analyses of the sulfated modification as well as level of KS are required to elucidate the underlying pathogenic mechanisms.

## Congenital Disorders Caused by Deficiency of Uridine Diphosphate-Glucuronic Acid and 3′-Phosphoadenosine 5′-Phosphosulfate Synthase

### Developmental and Epileptic Encephalopathy Caused by Mutations in *UGDH*

*UGDH* encodes UDP-Glc dehydrogenase (UGDH), which is an oxidoreductase that converts UDP-Glc to UDP-GlcA in the cytosol (Figure 1; Spicer et al., 1998). Compound heterozygous and homozygous mutations in *UGDH* cause developmental and epileptic encephalopathy, characterized by intractable epileptic seizures and developmental delay (Table 5; Freeze et al., 2015; McTague et al., 2016; Hengel et al., 2020). The recombinant mutant enzyme of UGDH, p.Ala44Val and p.Ala82Thr, mainly forms a dimer or remains a monomer species, respectively, whereas the wild-type UGDH predominantly forms a hexamer (Hengel et al., 2020). Furthermore, the values of *V*_*max*_ of p.Ala44Val and p.Ala82Thr enzymes were less than 50% and at an undetermined level, respectively, compared with that of the wild-type (Hengel et al., 2020). The probands of 36 cases from 25 families showed epileptic encephalopathy with developmental delay and hypotonia (Hengel et al., 2020). These hallmarks are similar to those probands with mutations in *CHSY1*, *EXTL3*, or *NDST1* (Li et al., 2010; Tian et al., 2010; Najmabadi et al., 2011; Reuter et al., 2014; Guo et al., 2017b; Oud et al., 2017; Volpi et al., 2017). Thus, UGDH and/or UDP-GlcA is responsible for not only neuronal development but also the biosynthesis of GAGs. Although the level of GAGs in patients with mutations in *UGD*H has not been investigated, the embryos from mutant mice, *lazy mesoderm*, with mutations in *UGDH* showed less staining with anti-CS or anti-HS antibodies, compared with the wild-type embryos for both antibodies (García-García and Anderson, 2003). Further analyses including quantification of GAGs from patients and glucuronokinase as well as UDP-GlcA pyrophosphrylase that form GlcA-1-phosphate and UDP-GlcA, respectively, which have been identified in zebrafish but not mammals, are required.

**TABLE 5:** Congenital disorders of UDP-sugars, PAPS, and related protein deficiencies*.

Gene	Protein	Chromosomal location	MIM number	Name of disorder	Clinical hallmarks
*UGDH*	UDP-Glc dehydrogenase		603370 618792	Developmental and epileptic encephalopathy 84	Intractable epileptic seizures and developmental delay.
*PAPSS2*	PAPS synthase 2	10q23.2-q23.3	612847 603005	Brachyolmia 4 with mild epiphyseal and metaphyseal changes; Spondyloepimetaphyseal dysplasia Pakistani type (PAPSS2 type); Hyperandrogenism	Short, bowed lower limbs, enlarged knee joints, kyphoscoliosis, and mild generalized brachydactyly, androgen excess, premature pubarche, hyperandrogenic anovulation, dehydroepiandrosterone, a short trunk.
*SLC26A2*	Diastrophic dysplasia sulfate transporter (DTDST)	5q32	600972 256050 222600 226900 606718	Achondrogenesis type IB; Atelosteogenesis type II; De la Chapelle dysplasia; Diastrophic dysplasia; Diastrophic dysplasia, broad bone-platyspondylic variant; Epiphyseal dysplasia multiple 4	Lethal chondrodysplasia with severe under-development of the skeleton, extreme micromelia, death before or immediately after birth, epiphyseal dysplasia and early-onset osteoarthritis.
*SLC35A3*	UDP-GlcNAc transporter	1p21.2	605632 615553	Arthrogryposis, intellectual disability, and seizures	Arthrogryposis, knee and hip dislocations, anomalous vertebrae, hypotonia, autism, epilepsy, seizure, and mild to moderate intellectual disability.
*SLC35D1*	UDP-GlcA/UDP-GalNAc dual transporter	1p31.3	610804 269250	Schneckenbecken dysplasia	Neonatal lethal chondrodysplasia, platyspondyly with oval-shaped vertebral bodies, extremely short long bones with a dumbbell-like appearance, and small ilia with a snail-like appearance.
*CANT1*	UDP diphosphatase	17q25.3	617719 251450 613165	Desbuquois dysplasia 1; Epiphyseal dysplasia multiple 7	A short stature, joint laxity, scoliosis, and advanced carpal ossification with a delta phalanx.
			264180	Pseudodiastrophic dysplasia	See disorder of *B3GAT3* in Table 1.
*IMPAD1*	3′-Phosphoadenosine 5′-phosphate 3′-phosphatase	8q12.1	614078 614010	Chondrodysplasia with joint dislocations GRAPP type	A short stature, chondrodysplasia, with brachydactyly, congenital joint dislocations, a cleft palate, and facial dysmorphism.
*GORAB*	Golgin, Rab6-interacting protein	1q24.2	607983 231070	Geroderma osteodysplasticum	Lax and wrinkled skin, joint laxity, a typical face with a prematurely aged appearance, severe osteoporosis, malar and mandibular hypoplasia, and a variable degree of growth retardation.
*COG4*	Component of oligomeric Golgi complex 4	16q22.1	606976 618150	Saul-Wilson syndrome	A short stature, prominent forehead, prominent eyes, narrow nasal bridge, micrognathia, clubfoot, brachydactyly, short distal phalanges of fingers, irregular end plates of vertebral bodies, hypoplasia of the odontoid process, and metaphyseal flaring in the long bones.

*, the table was cited from a reference (Mizumoto, 2018) with modifications. SLC, solute carrier family; CANT1, calcium-activated nucleotidase; IMPAD1, inositol monophosphatase domain-containing protein 1.

### Spondyloepimetaphyseal Dysplasia of the Pakistani Type and Brachyolmia 4 With Mild Epiphyseal and Metaphyseal Changes Caused by Mutations in *PAPSS2*

*PAPSS2* encodes PAPS synthase 2 (PAPSS2), which is a dual enzyme containing ATP sulfurylase and adenosine 5′-phosphosulfate kinase domains at C- and N-terminals, respectively (Figure 1; Fuda et al., 2002; Venkatachalam, 2003). Spondyloepimetaphyseal dysplasia of the Pakistani type that is characterized by a short stature at birth, short-bowed lower limbs, mild brachydactyly, kyphoscoliosis, enlarged knee joints, and early-onset degenerative joint disease of the hands and knees, is caused by homozygous mutations in *PAPSS2* (Table 5; Ahmad et al., 1998; Tüysüz et al., 2013).

Compound heterozygous mutations in *PAPSS2* cause hyperandrogenism, premature pubarche, and hyperandrogenic anovulation, in addition to a short stature and skeletal dysplasia, which may result from a low level of serum dehydroepiandrosterone sulfate with increasing androgen (Table 5; Noordam et al., 2009).

Furthermore, brachyolmia, which is characterized by a short stature, short trunk, irregular endplates, narrow intervertebral disks, precocious calcification of rib cartilages, a short femoral neck, mildly shortened metacarpals, and a normal intelligence and facies, are also caused by compound heterozygous and homozygous mutations in *PAPSS2* (Table 5; Miyake et al., 2012; Iida et al., 2013; Bownass et al., 2019). *PAPSS2* mutation might present a gradation of the phenotypic spectrum from brachyolmia to spondylo-epiphyseal and spondylo-epimetaphyseal dysplasia of the Pakistani type (Miyake et al., 2012). Brachymorphic mice, which have a missense mutation, p.Gly79Arg, in *PAPSS2*, are characterized by short stature as well as low level of CS (Sugahara and Schwartz, 1979; Pennypacker et al., 1981; Kurima et al., 1998). These findings indicate that sulfated modification of CS is required for cartilage development.

## Congenital Disorders by Deficiency of Uridine Diphosphate-Sugar and Sulfate Transporters

### Diastrophic Dysplasia and Achondrogenesis Type IB Caused by Mutations in *SLC26A2*

*SLC26A2* encodes a sulfate transporter, which incorporates sulfate anions into the cytosol at the plasma membrane (Figure 1; Hästbacka et al., 1994; Satoh et al., 1998; Seidler and Nikolovska, 2019). The incorporated sulfate is activated to adenosine-phosphosulfate and then to PAPS by PAPS synthase (Venkatachalam, 2003). Mutations in *SLC26A2* cause diastrophic dysplasia, achondrogenesis 1B, and atelosteogenesis type 2 (Table 5; Hästbacka et al., 1994, 1996; Superti-Furga et al., 1996; Rossi and Superti-Furga, 2001). Diastrophic dysplasia is an osteochondrodysplasia characterized by a short-limbed short stature, kyphoscoliosis, generalized dysplasia of the joints, hitchhiker thumbs, metatarsus adductus deformity of the feet, deformation of the ear lobes, and cleft plate (Superti-Furga et al., 1996). Achondrogenesis 1B is also a recessively inherited chondrodysplasia, which is characterized by extremely poor skeletal development and perinatal death (Table 5; Superti-Furga et al., 1996). Furthermore, atelosteogenesis type 2 is characterized by severely shortened limbs, a small chest, scoliosis, a club foot, abducted thumbs and great toes, and cleft palate (Table 5; Hästbacka et al., 1996). The incorporation of sulfate and formation of adenosine-phosphosulfate as well as PAPS were impaired in cultured cells from the proband of achondrogenesis 1B, thereby resulting in the sulfation of PGs (Superti-Furga et al., 1996). These findings suggest that mutations in *SLC26A2* cause low sulfation of GAGs, thereby disturbing the assembly of the extracellular matrix as well as cellular signaling.

### Arthrogryposis, Intellectual Disability, and Seizures Caused by Mutations in *SLC35A3*

*SLC35A3* encodes UDP-GlcNAc transporter, which incorporates UDP-GlcNAc into the Golgi apparatus from the cytosol (Figure 1; Ishida et al., 1999; Szulc et al., 2020). Compound heterozygous and homozygous mutations of *SLC35A31* cause neuroskeletal disorder, which is characterized by arthrogryposis, knee and hip dislocations, anomalous vertebrae, hypotonia, autism, epilepsy, seizure, and mild to moderate intellectual disability (Table 5; Edvardson et al., 2013; Edmondson et al., 2017; Miyake et al., 2021). Moreover, the probands showed deficiency of several N-glycans, no difference in the biosynthesis of keratan sulfate, and hallmarks such as cleft palate, micrognathia, a patent foramen ovale, patent ductus arteriosus, posterior embryotoxon, short limbs, camptodactyly, talipes valgus, rocker-bottom feet, and facial dysmorphism (Edvardson et al., 2013; Edmondson et al., 2017). Notably, a CS side chain of serum bikunin, CS-PG, from a patient with mutation in *SLC35A3* was shorter than that from a control subject (Haouari et al., 2020). Because CS did not contain the GlcNAc residue, the finding indicates several possibilities that SLC35A3 transports UDP-GalNAc in addition to UDP-GlcNAc, and that UDP-GlcNAc-4′-epimerase/UDP-Gal-4′-epimerase encoded by *GALE* is located in the Golgi apparatus in addition to cytosol (Thoden et al., 2001; Broussard et al., 2020; Fushinobu, 2021).

### Schneckenbecken Dysplasia Caused by Mutations in *SLC35D1*

*SLC35D1* encodes UDP-GlcA/UDP-GalNAc dual transporter, which incorporates UDP-GlcA and UDP-GalNAc into the Golgi apparatus from the cytosol (Figure 1; Muraoka et al., 2001). Mutations of *SLC35D1* cause Schneckenbecken dysplasia, which is characterized by perinatally lethal skeletal dysplasia, thoracic hypoplasia, severe flattening of the vertebral bodies and short, thick long bones (Table 5; Hiraoka et al., 2007; Furuichi et al., 2009). The German term “Schneckenbecken” refers to the snail-like appearance of the ilia that results from a medial bone projection from the inner iliac margin (Nikkels et al., 2001). Mice deficient in *Slc35d1^–/–^* showed decreased CS in the cartilage and no change in HS (Hiraoka et al., 2007). These findings suggest that SLC35D1 predominantly transports UDP-GalNAc rather than UDP-GlcA *in vitro* to form CS polysaccharides in the Golgi apparatus.

## Congenital Disorders Caused by Glycosaminoglycan-Related Genes

### Desbuquois Dysplasia 1 Caused by Mutations in *CANT1*

*CANT1* encodes a calcium-dependent nucleoside diphosphatase that hydrolyzes UDP, which is a reaction product after transfer of a sugar residue by a glycosyltransferase, into uridine 5′-monophosphate (UMP) and an inorganic phosphate in the Golgi apparatus (Figure 1; Failer et al., 2002; Smith et al., 2002). The compound heterozygous and homozygous mutations cause Desbuquois dysplasia type 1, which is characterized by severe growth retardation, joint laxity, short extremities, and progressing scoliosis (Table 5; Huber et al., 2009; Faden et al., 2010; Dai et al., 2011; Furuichi et al., 2011; Laccone et al., 2011; Nizon et al., 2012b; Singh et al., 2015; Balasubramanian et al., 2017; Menzies et al., 2019). The phenotypes of this disorder are common to those of Desbuquois dysplasia type 2 caused by mutations in *XYLT1* (Bui et al., 2014; Silveira et al., 2016; Al-Jezawi et al., 2017; Guo et al., 2017a).

Homozygous mutation in *CANT1* causes pseudodiastrophic dysplasia, which is associated with prenatal manifestation and early lethality (Table 5; Byrne et al., 2020). The probands showed similar hallmarks with the probands with a compound heterozygous mutation in *B3GAT3* (Byrne et al., 2020).

These findings suggest that the accumulation of UDP in the Golgi apparatus might inhibit the enzyme activity of XYLT1, B3GAT3 and UDP-GlcA decarboxylase/UDP-Xyl synthase, and the decrease in UMP might lead to impairment of UDP-Xyl or UDP-GlcA transporters, which are antiporters for UMP and UDP-XYl or UDP-GlcA, respectively, thereby resulting in defects in the biosynthesis of GAGs.

### Chondrodysplasia With Joint Dislocations of GPAPP Type Caused by Mutations in *IMPAD1*

The sulfate donor substrate, PAPS, is converted into adenosine-3′, 5′-bisphosphate after a reaction with a sulfotransferase in the Golgi apparatus. *IMPAD1* encodes inositol monophosphatase domain-containing protein 1/Golgi 3′-phosphoadenosine 5′-phosphate 3′-phosphatase (IMPAD1/GPAPP), which hydrolyzes the phosphate from 3′-phosphoadenosine 5′-phosphate to adenosine 5′-phosphate (Figure 1; Vissers et al., 2011; Nizon et al., 2012a). Homozygous mutations in *IMPAD1* cause chondrodysplasia with joint dislocations (Table 5; Vissers et al., 2011; Nizon et al., 2012a), which is characterized by a short stature, brachydactyly, joint dislocations, micrognathia, cleft palate, and facial dysmorphism (Vissers et al., 2011; Nizon et al., 2012a). The knockout mice, *Impad1*^–/–^, exhibited a reduction of 4-*O*-sulfated CS with increasing non-sulfated CS, and a small decrease in total sulfated HS with a corresponding increase of non-sulfated HS in the lung (Frederick et al., 2008). Therefore, the catabolic abnormality in the reaction product of donor substrate including 3′-phosphoadenosine 5′-phosphate may result in the inhibition of GAG-sulfotransferases, and such skeletal disorders develop.

### Geroderma Osteodysplasticum Caused by Mutations in *GORAB*

*GORAB* encodes Rab6-interacting Golgi protein (Hennies et al., 2008; Takeda et al., 2017). The homozygous mutations in *GORAB* cause gerodermia osteodysplastica that is characterized by skin laxity and early-onset osteoporosis (Table 5; Rajab et al., 2008). The knockout mice, *GORAB*^–/–^, showed a reduction in the level of DS, but not CS and HS, in skin and cartilage (Chan et al., 2018). These findings suggest that the loss of *Gorab* results in a defect of the DS side chain in PGs due to disturbed transport in the Golgi compartment, leading to the development of gerodermia osteodysplastica.

### Saul-Wilson Syndrome Caused by Mutations in *COG4*

*COG4* encodes the component of oligomeric Golgi complex 4, which is involved in intracellular vesicular transport (Climer et al., 2018). The heterozygous mutation in *COG4* causes a rare skeletal dysplasia, Saul-Wilson syndrome, which is characterized by a short stature, prominent forehead, prominent eyes, a narrow nasal bridge, micrognathia, clubfoot, brachydactyly, short distal phalanges of fingers, irregular end plates of vertebral bodies, hypoplasia of the odontoid process, and metaphyseal flaring in the long bones (Table 5; Saul and Wilson, 1990; Ferreira et al., 2018). Although protein *N*-glycosylation in sera and fibroblasts from probands was not notably altered, DS-PG, decorin, was affected. A smaller population of glycanated decorin and longer CS/DS side chain on decorin in the affected fibroblasts were observed, compared with those in healthy subjects (Ferreira et al., 2018). These findings suggest that specific transport by oligomeric Golgi complex might affect the cellular localization and/or polymerization as well as sulfation of specific PGs.

## Congenital Disorders Caused by Mutations in Core Proteins of Proteoglycans

### Chondroitin Sulfate-Proteoglycans

Aggrecan encoded by *ACAN* consists of a protein with 2,431 amino acids (∼220 kDa), which is modified by CS (>100 chains per monomer), KS, and other oligosaccharides (Doege et al., 1991). The autosomal dominant spondyloepiphyseal dysplasia Kimberley type, which is characterized by short stature, stocky build, early-onset osteoarthritis of joints, flattened vertebral bodies with sclerosis and end-plate irregularity, and flattened femoral epiphyses, is caused by a single–base-pair insertion in *ACAN*, which introduces a frameshift of 212 amino acids, including 22 cysteine residues, followed by a premature stop codon (Table 6; Gleghorn et al., 2005). Furthermore, homozygous or heterozygous mutations in *ACAN* cause spondyloepimetaphyseal dysplasia Aggrecan type or dominant familial osteochondritis dissecans, respectively (Tompson et al., 2009; Stattin et al., 2010).

**TABLE 6:** Congenital disorders of core proteins of PGs.

Gene	Protein	Chromosomal location	MIM number	Name of disorder	Clinical hallmarks
*ACAN*	Aggrecan	15q26.1	155760 608361 165800 612813	Spondyloepiphyseal dysplasia Kimberley type; Spondyloepimetaphyseal dysplasia, aggrecan type; Short stature and advanced bone age, with or without early-onset osteoarthritis and/or osteochondritis dissecans	A short stature, stocky build, early-onset osteoarthritis of joints, flattened vertebral bodies with sclerosis and end-plate irregularity, and flattened femoral epiphyses.
*VCAN*	Versican	5q14.2-q14.3	118661 143200	Wanger syndrome	Vitreoretinopathy, an empty vitreous cavity, vitreous degeneration, progressive chorioretinal atrophy, perivascular sheathing, subcapsular cataract, and myopia.
*BGN*	Biglycan	Xq28	301870 300106 300989	Spondyloepimetaphyseal dysplasia, X-linked; Meester-Loeys syndrome	Anomalies of spine and epiphyses, metaphyses of long bones, a short stature, and osteoarthritic changes in joints
*DCN*	Decorin	12q21.33	125255 610048	Corneal dystrophy, congenital stromal	Corneal clouding, refractive errors, amblyopia, strabismus, nystagmus, and esotropia.
*GPC3*	Glypican 3	Xq26.2	300037 312870	Simpson-Golabi-Behmel syndrome type 1	Pre- and postnatal overgrowth, visceral and skeletal anormalies, a coarse face, heart defects, and hypotonia.
*GPC4*	Glypican 4	Xq26.2	300168 301026	Keipert syndrome	Craniofacial and digital abnormalities, variable learning difficulties, and sensorineural deafness.
*GPC6*	Glypican 6	13q31.3-q32.1	604404 258315	Omodysplasia 1	Proximally shortened limbs, facial dysmorphism, a severely short stature, and hypoplastic humeri.
*HSPG2*	Perlecan	1p36.12	255800 142461	Schwartz-Jampel syndrome	Permanent myotonia and skeletal dysplasia including a short stature, kyphoscoliosis, bowing of the diaphyses and irregular epiphyses.
			224410	Dyssegmental dysplasia Silverman-Handmaker type	A flat face, micrognathia, cleft palate, reduced joint mobility, and encephalocele.
*AGRN*	Agrin	1p36.33	103320 615120	Myasthenic syndrome, congenital, 8, with pre- and post-synaptic defects	Changes in the nerve-terminal cytoskeleton and fragmentation of the synaptic gutters, and muscle weakness.
*COL18A1*	Collagen 18 α1	21q22.3	120328 267750	Knobloch syndrome type 1	High myopia, vitreoretinal degeneration with retinal detachment, macular abnormalities, and occipital encephalocele.
*KERA*	Keratocan	12q21.33	603288 217300	Cornea plana 2, autosomal recessive	Reduced corneal curvature, hyperopia, hazy corneal limbus, and arcus lipoides.

Versican encoded by *VCAN* consists of a protein with 3,396 amino acids, which has ∼20 putative CS attachment sites as well as potential *N*- and *O*-glycosylation sites (Dours-Zimmermann and Zimmermann, 1994). The autosomal dominant Wanger syndrome, which is characterized by vitreoretinopathy, an empty vitreous cavity, vitreous degeneration, progressive chorioretinal atrophy, perivascular sheathing, subcapsular cataract, and myopia, is caused by heterozygous mutations in *VCAN* (Table 6; Miyamoto et al., 2005; Kloeckener-Gruissem et al., 2006, 2013).

### Dermatan Sulfate-Proteoglycans

Biglycan encoded by *BGN* consists of a protein with 368 amino acids, which has 2 putative DS attachment sites (Fisher et al., 1989). Two missense mutations in *BGN* cause the X-linked form of spondyloepimetaphyseal dysplasia, which is characterized by anomalies of the spine as well as the epiphyses and metaphyses of the long bones, thereby resulting in a short stature and osteoarthritic changes in joints (Table 6; Cho et al., 2016). Thoracic aortic aneurysms and dissections are also caused by missense and non-sense mutations and 21 or 28-kb deletions in *BGN* (Meester et al., 2017). The clinical hallmarks are characterized by the early-onset of aortic aneurysm and dissection, joint hypermobility and contractures, deformities of the skin striae and pectus, craniofacial dysmorphisms including dolichocephaly, hypertelorism, down-slanting eyes, a high-arched plate, proptosis, malar hypoplasia, and frontal bossing as well as manifestations of Loeys-Dietz syndrome such as a bifid uvula and cervical spine instability (Meester et al., 2017). Moreover, TGFβ signaling is up-regulated in patients, thereby being similar to probands with Loeys-Dietz syndrome caused by mutations in TGFβ signaling-related proteins (Van Laer et al., 2014).

Decorin encoded by *DCN* consists of a protein with 359 amino acids, which has a putative DS attachment site (Fisher et al., 1989). Autosomal dominant congenital stromal corneal dystrophy is caused by mutations in *DCN*, and is characterized by corneal clouding with fine opacities observed as small flakes and spots, refractive errors, amblyopia, strabismus, nystagmus, and esotropia (Table 6; Van Ginderdeuren et al., 2002; Bredrup et al., 2005; Rødahl et al., 2006; Kim et al., 2011; Jing et al., 2014). All mutations in *DCN* causing dominant corneal dystrophy result in the truncated forms of decorin core protein, which may affect matrix assembly in the corneal stroma in a dominant-negative manner, supported by analyses using model mice with a frameshift mutation in *Dcn* (Chen et al., 2011).

### Heparan Sulfate-Proteoglycans

Glypicans encoded by *GPC1* to *GPC6* consist of proteins with 550–580 amino acids, which have two putative HS attachment sites at the C-terminus. Glypicans are bound to the plasma membrane by a glycosylphosphatidylinositol (GPI) anchor (Filmus et al., 2008; Filmus and Capurro, 2014). Simpson-Golabi-Behmel syndrome is an X-linked syndrome, caused by mutations in *GPC3*, and characterized by pre- and postnatal overgrowth, visceral and skeletal anormalies, a coarse face, heart defects, and hypotonia (Table 6; Veugelers et al., 2000; Sakazume et al., 2007). Keipert syndrome, known as nasodigitoacoustic syndrome, is also an X-linked syndrome, caused by mutations in *GPC4*, and characterized by craniofacial and digital abnormalities, variable learning difficulties, and sensorineural deafness (Table 6; Amor et al., 2019). Autosomal-recessive omodysplasia is caused by homozygous mutations in *GPC6*, and is characterized by proximally shortened limbs, facial dysmorphism, a severely short stature, and hypoplastic humeri (Table 6; Campos-Xavier et al., 2009).

Perlecan encoded by *HSPG2* consists of a protein with 4,392 amino acids (∼467 kDa), which is a major HS-PG with two or three HS as well as one or two CS, distributed to the basement membrane as well as the extracellular matrix of muscle, cartilage, and bone marrow (Cohen et al., 1993; Nicole et al., 2000; Melrose, 2020). Schwartz-Jampel syndrome is an autosomal recessive disorder, caused by homozygous or compound heterozygous mutations in *HSPG2*, and characterized by permanent myotonia and skeletal dysplasia including a short stature, kyphoscoliosis, bowing of the diaphysis, and irregular epiphyses (Table 6; Nicole et al., 2000; Arikawa-Hirasawa et al., 2002; Stum et al., 2006). Dyssegmental dysplasia Silverman-Handmaker type is an autosomal recessive skeletal dysplasia with anisospondyly and micromelia, and is caused by homozygous or heterozygous mutations in *HSPG2* (Table 6; Arikawa-Hirasawa et al., 2001). The probands showed the hallmarks including a flat face, micrognathia, cleft palate, reduced joint mobility, and encephalocele.

Agrin encoded by *AGRN* consists of a protein with 2,068 amino acids, which has three potential HS attachments sites, and an extracellular matrix molecule released by the nerve and critical for formation of the neuromuscular junction (Rupp et al., 1991, 1992; Denzer et al., 1995). Myasthenic syndrome is a neuromuscular transmission disorder characterized by a major disorganization of the neuromuscular junction such as changes in the nerve-terminal cytoskeleton and fragmentation of the synaptic gutters, thereby leading to muscle weakness, caused by homozygous or heterozygous mutations in *AGRN* (Table 6; Huzé et al., 2009; Maselli et al., 2012).

Collagen18α1 encoded by *COL18A1* consists of a protein with 1,339 amino acids, which is a subfamily of collagen predominantly expressed in the extracellular matrix of the liver, lung, and kidney, and is a subfamily of collagen distributed in the extracellular matrix (Oh et al., 1994). Knobloch syndrome type 1 is an autosomal recessive disorder defined by the occurrence of high myopia, vitreoretinal degeneration with retinal detachment, macular abnormalities, and occipital encephalocele, which is caused by homozygous or compound heterozygous mutations in *COL18A1* (Table 5; Sertié et al., 2000; Suzuki et al., 2002; Caglayan et al., 2014).

### Kepatan Sulfate-Proteoglycans

Keratocan encoded by *KERA* consists of a protein core of 352 amino acids with three covalent KS attachment sites at asparagine residues (Corpuz et al., 1996). Cornea plana type 2 is autosomal recessive and a more severe disease than type 1, characterized by a reduced corneal curvature, hyperopia, hazy corneal limbus, and arcus lipoides, and is caused by homozygous mutations in *KERA* (Table 6; Tahvanainen et al., 1996; Pellegata et al., 2000).

## Conclusion and Perspectives

Genomic sequences have led to the identification of a variety of congenital disorders caused by defects in anabolism and catabolism of GAGs as well as PGs. Some of them have common clinical features such as skeletal, skin, heart, neuronal, and immune abnormalities. However, the molecular mechanisms of the onsets underlying these congenital diseases remain to be elucidated. To clarify the molecular pathogeneses including analyses of the functional domains of GAGs, identifications of the affected core proteins of PGs, the functional proteins interacting with GAGs, and the signaling pathways transduced by GAGs, are required. Achievement of these analyses lead to new therapeutics including the applications of adeno-associated virus, enzyme-replacement therapy, oligosaccharides derived from GAGs, and mimetics of GAGs for these disorders.

## Author Contributions

SM wrote the manuscript. SY provided fruitful comments. Both authors contributed to the article and approved the submitted version.

## Conflict of Interest

The authors declare that the research was conducted in the absence of any commercial or financial relationships that could be construed as a potential conflict of interest. The handling editor declared a past co-authorship with the authors SM and SY.

## Publisher’s Note

All claims expressed in this article are solely those of the authors and do not necessarily represent those of their affiliated organizations, or those of the publisher, the editors and the reviewers. Any product that may be evaluated in this article, or claim that may be made by its manufacturer, is not guaranteed or endorsed by the publisher.
